# The COVID-19 Pandemic: Disproportionate Thrombotic Tendency and Management Recommendations

**DOI:** 10.3390/tropicalmed6010026

**Published:** 2021-02-18

**Authors:** Sabina Karim, Amin Islam, Shafquat Rafiq, Ismail Laher

**Affiliations:** 1 Department of Paediatric Haematology and Oncology, National Institute of Cancer Research and Hospital, Mohakhali, Dhaka 1212, Bangladesh; 2 Mid and South Essex University Hospitals Group NHS Trust, Westcliffe on Sea, Prittlewell Chase SS0 0RY, UK; amin.islam@qmul.ac.uk; 3 Department of Haematology and Oncology, Faculty of Medicine and Dentistry, Queen Mary University, London E1 4NS, UK; 4 Anglia Ruskin Medical School, Bishop Hall Ln, Chelmsford CM1 1SQ, UK; 5 Department of Gastroenterology, East Kent University Hospitals NHS Trust, Kennington Rd, Willesborough, Ashford TN24 0LZ, UK; srafiq@outlook.com; 6 Faculty of Medicine, Department of Anesthesiology, Pharmacology and Therapeutics, The University of British Columbia, 2176 Health Sciences Mall, Vancouver, BC V6T 1Z3, Canada; ismail.laher@ubc.ca

**Keywords:** anticoagulant, antiplatelet, antithrombotic therapy, COVID-19, SARS-CoV-2, thrombosis, disseminated intravascular coagulation

## Abstract

COVID-19 is an infectious disease caused by the SARS COV-2 virus. Patients with COVID-19 are susceptible to thrombosis due to excessive inflammation, platelet activation, endothelial dysfunction, and circulatory stasis, resulting in an increased risk of death due to associated coagulopathies. In addition, many patients receiving antithrombotic therapy for pre-existing thrombotic diseases can develop COVID-19, which can further complicate dose adjustment, choice and laboratory monitoring of antithrombotic treatment. This review summarizes the laboratory findings, the prohemostatic state, incidence of thromboembolic events and some potential therapeutic interventions of COVID-19 associated coagulopathy. We explore the roles of biomarkers of thrombosis and inflammation according to the severity of COVID-19. While therapeutic anticoagulation has been used empirically in some patients with severe COVID-19 but without thrombosis, it may be preferable to provide supportive care based on evidence-based randomized clinical trials. The likely lifting of travel restrictions will accelerate the spread of COVID-19, increasing morbidity and mortality across nations. Many individuals will continue to receive anticoagulation therapy regardless of their location, requiring on-going treatment with low-molecular weight heparin, vitamin K antagonist or direct-acting anticoagulants.

## 1. Background

Travel has greatly accelerated the global spread of COVID-19 and has so far affected over 107 million people with more than 2.3 million deaths (as of 10 February 2021) [1]. SARS-CoV-2, the cause of the COVID-19 pandemic, replicates in the upper respiratory tract to enable active viral shedding with minimal symptoms [2]. Survival of the virus for 24 to 72 h on different types of surfaces further facilitates fomite transmission [3], allowing the virus to be readily transmissible in travel settings. The early symptoms of COVID-19, such as fever, fatigue, headache, cough, shortness of breath, diarrhea and myalgia, are similar to those in other viral infections [4]. The virus binds to the angiotensin converting enzyme 2 (ACE2) receptor, which is expressed at higher levels in males compared to females, and also in Asians compared to white Caucasians or Africans [5]. The clinical course of the disease can be divided into three phases: the viremic phase, the acute or pneumonic phase and the severe or recovery phase [6]. Much like other virulent zoonotic coronavirus infections such as severe acute respiratory syndrome (SARS) and Middle East respiratory syndrome corona virus (MERS-CoV), COVID-19 can potentially lead to systemic inflammatory response syndrome (SIRS), acute respiratory distress syndrome (ARDS), multi-organ dysfunction and shock [7]. Though severe COVID-19 and its complications are common in the elderly and individuals with comorbidities such as diabetes and cardiovascular diseases, younger and healthy persons are not always spared and can also develop severe and complicated disease [8]. Increases in lactate dehydrogenase, C-reactive protein (CRP), D-dimer, ferritin and interleukin-6 (IL-6) are common laboratory findings in patients with COVID-19 [4,9]. Plasma IL-6 levels can correlate with disease severity and pro-coagulant states [9].

## 2. Possible Pathophysiology of Coagulopathy

**Figure 1:**
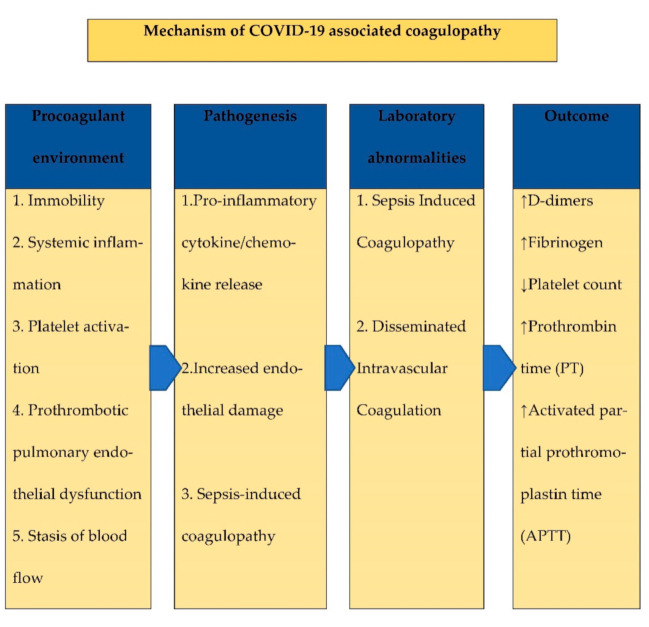
Mechanisms of COVID-19 associated coagulopathy.

There is a complex interplay between pro-inflammatory cytokine/chemokine release, increased endothelial dysfunction/damage and potential sepsis-induced coagulopathy during the acute phase of the disease, which in severe cases can increase the risk of thrombosis (Figure 1). Increased pro-thrombotic characteristics of COVID-19 likely results from (a) severe and prolonged hypoxemia that stimulates thrombosis, (b) cytokine storms in critically ill patients, and (c) a presumed role of local pulmonary thrombotic phenomena. It is presumed that prothrombotic pulmonary endothelial dysfunction leads to severe acute inflammation (through release of complement and cytokines) and blood coagulation activation with vascular microthrombosis that triggers further coagulopathy, leading to disseminated intravascular coagulation (DIC) [10].

Post-mortem histological similarities suggest that Severe Acute Respiratory Syndrome-Coronavirus (SARS-CoV), the cause of a previous endemic between 2002 and 2003, also causes ARDS with visible localized pulmonary hemorrhage, pulmonary oedema, desquamation with hyaline membrane formation and interstitial mononuclear inflammatory infiltrates. Localized pulmonary arteriolar thrombosis observed with SARS has not yet been described in autopsy reports of patients with COVID-19 [11]. Pulmonary vasculature thrombosis is likely to result from severe hypoxia, which is a powerful stimulant of coagulation.

## 3. Biomarkers of Hemostasis

Thrombocytopenia and increased D-dimer levels are consistently associated with an increased need for mechanical ventilation, admission to the intensive care unit (ICU) or death [12,13]. The severity of COVID-19 is frequently associated with prolongations of prothrombin time (PT), international normalized ratio (INR) and thrombin time (TT), and a trend of increases in activated partial thromboplastin time (aPTT) [14,15,16]. Retrospective analysis of hospitalized patients with COVID-19 indicates high levels in D-dimers and fibrin degradation products, prolonged PTs and aPTT in non-survivors compared to survivors. It is estimated that 71% of patients succumbing to the complications of COVID-19 met the International Society on Thrombosis and Haemostasis (ISTH) criteria for DIC, compared to just 0.6% for survivors [17,18].

## 4. Potential Role of Complement Inhibition in COVID-19

Thrombotic microangiopathy (TMA) can occur in many different clinical conditions, including pathogenic complement activation. The complement system mediates the innate immune response that promotes inflammation, defends against bacterial infections, and often neutralizes infectious viruses [19]. Two murine studies investigated complement activation in coronavirus infections to determine whether activation of the system could be protective or pathogenic. In a murine model lacking C3 and unable to activate the common complement pathway, SARS-CoV infection severity was decreased with less respiratory dysfunction and lower cytokine levels despite equal viral loads [20], suggesting that a significant portion of SARS-mediated disease is likely immune mediated. There were increased concentrations of C5a and C5b-9 in sera and lung tissues in a mouse model of MERS-CoV infection [21]. Blocking C5a with an antibody alleviated lung and spleen damage, with decreased cytokine response and viral replication. Evidence is emerging that the complement system is overactivated in SARS-COV-2 as noted in previous coronavirus infections and this may play a central role in thrombosis and unbalanced immune response [22].

Excessive complement activation occurs in humans in a number of pathological settings, leading to diffuse TMA and end-organ dysfunction, e.g., atypical hemolytic-uremic syndrome (aHUS), a rare disorder of uncontrolled complement activation characterized by microangiopathic hemolytic anemia, thrombocytopenia and acute renal failure. TMA in aHUS results in renal dysfunction and, in rare cases, cardiac dysfunction. Importantly, aHUS is treatable with eculizumab, a C5 complement inhibitor. Early treatment with eculizumab can reverse both renal and cardiac dysfunction [23]. Although the use of complement inhibitors is limited to rare diseases, it should also be actively investigated in the treatment of COVID-19.

## 5. Role for Antivirals and Immunomodulatory Agents to Reduce the Development of Immunothrombosis

There are several potential control points in the pathophysiological cascade COVID-19, starting from the initial infection to later development of ARDS where targeted therapeutic interventions could reduce the severity of disease. There is a role for dexamethasone in the treatment of ARDS in moderate to severe COVID-19 infection; dexamethasone reduces mortality and has become the standard of care in addition to using anti-viral and immunomodulatory therapies. Excessive systemic inflammation in patients with severe COVID-19 is likely to deplete levels of Vit C, Vit D and Zn in many individuals. Several human and animal studies highlight the potential efficacy of supplementation with a combination of Zn, intravenous Vit C and oral Vit D. Inhibition of IL6 by tocilizumab shows beneficial effects in several clinical trials and could reduce microthrombosis. Moreover, when used at appropriate doses, these treatments generally have an exceptionally good safety record. Aspirin (acetylsalicylic acid), the macrolide antibiotic azithromycin, oral or intravenous administration of NAC (N-acetylcysteine) has a role in inhibiting NF-κB and reducing the activation of the coagulation cascade in severe cases of COVID-19 [24].

## 6. D-Dimer in COVID-19 and Coagulations Disturbances

Patient health can deteriorate rapidly in severe cases of COVID-19, leading to ARDS, septic shock, metabolic acidosis and coagulopathy including DIC. Levels of D-dimers, which originate from the breakdown of cross-linked fibrin and are related to activation of coagulation and fibrinolysis, are often markedly elevated in severe COVID-19 patients (Table 1) [25,26]. A retrospective cohort study of 191 patients reported that D-dimer levels greater than 1.0 μg/mL were associated with increased mortality (*p* = 0.0033) in patients with COVID-19 [8]. Levels of 2.0 μg/mL or more on admission were reported as the optimum cut-off for predicting in-hospital mortality for COVID-19 [27]. Nearly 90% of inpatients with pneumonia have increased coagulation activity as marked by elevated D-dimer levels. The levels of D-dimers on admission can be used to triage patients into critical care [8,14]. Increased D-dimer levels are associated with worse outcomes even though many patients may not have full blown DIC and have near normal levels of PT, aPTT and TT.

## 7. COVID-19, Elevated Troponin and Thrombotic Disease

Increased troponin levels in patients with COVID-19 are associated with poor outcomes [30], but the differential diagnosis for elevated troponin levels in patients with COVID-19 is broad [31] and ranges from nonspecific myocardial injury, impaired renal function (leading to troponin accumulation), myocarditis, pulmonary embolism (PE) and types 1 and 2 myocardial infarction (MI) [32,33]. Similarly, elevated natriuretic peptide levels is nonspecific [32] and consideration for thrombotic events such as PE should always be guided by clinical findings. Mortality rates are higher in patients with underlying cardiovascular disease due to COVID-19 infection [34]. Levels of high-sensitivity cardiac troponin I (hs-TnI) are useful in monitoring disease progression and mortality [35]. A retrospective study of hs-TnI levels and death in patients with COVID-19 (based on SARS-CoV-2 RNA detection) reported a univariable odds ratio of 80.1 (95% CI 10.3–620.4, *p* < 0.0001), which was higher compared to other biomarkers such as D-dimers and lymphocyte counts [8]. Another study of 416 hospitalized patients reported that hs-TnI was elevated in 20% of COVID-19 patients on presentation [36]. These patients were more likely to require invasive (22% vs. 4%, *p* < 0.001) or non-invasive (46% vs. 4%, *p* < 0.001) ventilation, develop complications such as ARDS (59% vs. 15%, *p* < 0.001) or acute kidney injury (9% vs. 0%, *p* < 0.001). Clinicians should remain alert that increased levels of hs-TnI can also be related to non-ischemic causes of myocardial injury and thereby avoid inappropriate use of other resources [37].

## 8. Venous Thromboembolism

Venous thromboembolism (VTE) is common in patients with COVID-19, although the prevalence remains unknown. A recent scoping review reported the incidence of VTE to be 20%, with a risk of stroke of 3%; both VTE and risk of stroke are increased in severely ill patients [38]. ARDS in patients with COVID-19 can cause hypoxic pulmonary vasoconstriction, pulmonary hypertension, and right ventricular failure; further injury from severe PE can be irreversible. Risk of VTE can be screened by levels of D-dimer and fibrinogen; a retrospective study suggests that D-dimer concentrations greater than 1.0 μg/mL predicted the risk of VTE [39]. Patients with one or more predisposing factors for VTE (such as being older, elevated CRP, increased D-dimers, high fibrinogen levels, tachypnea, fever, critical illness, infectious etiology and immobility) are at greater risk of such events during hospitalization and require close monitoring.

## 9. Management of VTE in Patients with COVID-19

Therapeutic anticoagulation is the mainstay of VTE management in patients either with or without COVID-19 [40,41,42]. Prescribing an anticoagulant agent should take into consideration underlying comorbidities; bleeding risk and the treatment choices can change during hospital stay or at discharge. Parenteral anticoagulation, for example with unfractionated heparin (UFH), is preferable in some inpatients with VTE as it can be temporarily withheld or reversed as no significant interactions have been reported with investigational COVID-19 therapies. However, using UFH has some disadvantages such as the variable times to achieve therapeutically activated partial thromboplastin time ratios and increased risks of infection to health care workers during frequent blood draws. Using low-molecular-weight heparin (LMWH) may be preferred in patients who are unlikely to need further procedures. Advantages of oral anticoagulation with direct oral anticoagulant therapy (DOACs) includes minimal monitoring, improved discharge planning and outpatient management, while potential disadvantages include clinical deterioration and an inability to access reversal agents in a timely manner. Use of DOACs or LMWH is preferable in patients who are ready for discharge as it can minimize contact with health care personnel during INR monitoring. Catheter driven reperfusion and thrombolysis therapy is often recommended for management of patients with an unstable and large PE, but many patients with COVID-19 can have absolute or relative contraindications (such as coagulopathy, thrombocytopenia, a recent invasive procedure, pericarditis, age > 75 years) to thrombolysis [43,44].

## 10. Outpatient Management with Mild COVID-19

Patients with mild symptoms of COVID-19 should stay at home and the routine use of thromboprophylaxis is not recommended; they should be assessed for potential risks of VTE or bleeding and should continue anticoagulant treatment for other indications. Such patients should be counselled on the transition to DOAC after considering the risks of bleeding, potential drug interactions, affordability and availability of drugs, and recent INR status. There may be limitations to monitoring INR at home or at nearby laboratories due to the risk of exposure to SARS-CoV-2. Patients not suitable for treatment with DOAC should use LMWH as a reasonable alternative. Patients with a stable INR and who did not require changes in dosage within the last six months can safely continue warfarin therapy [45].

## 11. Management of Hospitalized Patients with Moderate or Severe COVID-19 without DIC

Hospitalized patients with moderate to severe COVID-19 should be assessed for risks of VTE and DIC. Routine screening for VTE (e.g., with bilateral lower extremity ultrasound) in hospitalized patients with COVID-19 with elevated D-dimer levels (>1500 ng/mL) is not currently recommended. Signs of active bleeding should be monitored if DIC is suspected or confirmed. Every patient with moderate to severe symptoms should be offered thromboprophylaxis if not strictly contraindicated (for example, with severe thrombocytopenia, grossly deranged coagulation profiles or active bleeding). The choice of drugs, the dose and duration of treatment should follow national guidelines. Laboratory data monitoring, especially of D-dimers, should be checked every 2−3 days. Intermediate or therapeutic doses of thromboprophylaxis should be used at the discretion of the treating physician based on the risk of bleeding. A study of 92 ICU patients indicates a 21% overall rate of hemorrhagic events, of which nearly half (48%) received anticoagulation treatment [38]. Parenteral LMWH is the preferred choice for thromboprophylaxis due to its advantages related to dosing schedule and monitoring compared to intravenous heparin. Compliance is an important consideration in anticoagulant therapy; LMWH is administered mostly as a single daily dose, which improves compliance and thus outcomes. Drug interactions between antiviral treatments and DOACs, and the difficulty in maintaining stable INRs in patients prescribed vitamin K antagonists, means that LMWHs or UFH are preferable alternative treatments, either with or without mechanical prophylaxis.

## 12. Hospitalized Patients with Moderate or Severe COVID-19 and with Suspected or Confirmed DIC

Prophylactic anticoagulation should be administered to patients with moderate or severe COVID-19 diagnosed with DIC but without significant bleeding. There are currently insufficient data to consider routine therapeutic or intermediate-dose parenteral anticoagulation with UFH or LMWH in hospitalized patients with COVID-19 with suspected or confirmed DIC but with no overt bleeding. It is reasonable to consider the indications for anticoagulation therapy during dose adjustment or discontinuation in patients with moderate or severe COVID-19 already receiving chronic anticoagulation treatment and who develop suspected or confirmed DIC without overt bleeding. A common recommendation in such conditions is to reduce the dose of anticoagulant if the thrombotic risk is not excessive [45,46]. Patients with moderate or severe COVID-19 and receiving dual antiplatelet therapy (e.g., percutaneous coronary intervention within the past three months or recent myocardial infarction) should be assessed on an individual basis and serial platelet counts should be considered when making decisions on dose adjustments or discontinuation of treatment. In general, it is advisable to continue dual antiplatelet therapy if the platelet count is >50,000, reduce to single antiplatelet therapy if the platelet count is between 25,000 and 50,000, and discontinue antiplatelet therapy if the platelet count is below 25,000. These guidelines should be reviewed according to the risk of bleeding vs risk of thrombosis [17].

Risk assessment of VTE is reasonable when using pharmacological prophylaxis for up to 45 days post discharge. Pharmacological prophylaxis should be considered if there is an elevated risk for thrombotic events without a high bleeding risk. Patients should be counselled on the importance of ambulation and physical activity at home [45].

## 13. Patients with COVID-19 Presenting with Acute Coronary Syndrome (ACS)

Decisions regarding percutaneous coronary intervention or fibrinolytic therapy should be taken after assessing the severity of ST-elevation myocardial infarction (STEMI) and potential COVID-19 in patients and transmission risk to clinicians and healthcare providers [47].

## 14. Extended (Post-Discharge) VTE Prophylaxis

Post discharge extended thromboprophylaxis is recommended with LMWH or DOACs; even though these therapies reduce the risk of VTE, there remains the risk of bleeding events, including major bleeding [48,49,50,51,52,53,54]. Although there is little data specifically related to COVID-19, an individualized approach should be used after balancing the risks of hemorrhage and thrombosis, followed by extended prophylaxis (for up to 45 days) for patients at increased risk of VTE (e.g., reduced mobility, comorbidities such as active cancer, and elevated D-dimer levels more than twice higher than normal) but who are at a low risk of bleeding [50,55]. There is no clear guidance on thromboprophylaxis in patients quarantined with mild COVID-19 but having significant comorbidities, or for those without COVID-19 but who are less active because of quarantine measures. Such patients should be counselled about the importance of remaining physically active at home. Until more high-quality data are available, pharmacological prophylaxis should be reserved for patients with the highest risk, including those with limited mobility and a history of prior VTE or active malignancy.

## 15. Role for Empiric Therapeutic Anticoagulation without a Diagnosis of VTE

In view of the hemostatic derangements discussed above and from observations of other viral illnesses, some clinicians prefer the use of intermediate- or full-dose parenteral anticoagulation (rather than prophylactic dosing) for routine care of patients with COVID-19 based on the hypothesis that it could prevent microvascular thrombosis [56,57]. However, data to support this premise are primarily based on a subgroup analysis (n = 97) from a single retrospective study having limited control for potential confounders [17]. Another single-center study with 81 patients suggested that D-dimer levels greater than 1500 ng/mL have a sensitivity of 85.0% and specificity of 88.5% for detecting VTE events [58]. Many physicians prefer prophylactic anticoagulation treatment, while others consider the short-term use of intermediate or therapeutic doses as a reasonable approach. While physicians currently use a variety of prophylactic, intermediate, or therapeutic doses of anticoagulants in patients, the optimal dosing in patients with severe COVID-19 remains unknown.

## 16. Managing the Risk of Hospital-Associated VTE

Hospital-associated venous thromboembolism (HA-VTE) includes VTE presentation while hospitalized, and for up to 90 days post-discharge. Patients infected with COVID-19 are at increased risk of HA-VTE, especially if they become immobilized during critical care. It is unclear if hospitalized patients with COVID-19 are at increased risk for VTE compared to other patients with chest infections and elevated D-dimer values. Elevated D-dimer levels can also be used in a scoring system to identify those at increased risk of VTE [50,59]. Patients with severe COVID-19 are immobile, leading to an acute inflammatory state with a hypercoagulable state. There is also the possibility of endothelial cell activation/damage due to binding of the virus to ACE2 receptors [60].

## 17. COVID-19 and Interventional Therapies for VTE

The management of PE requires a multidisciplinary team [40,61,62,63]. It is important to note that there are limited data demonstrating lower mortality rates due to the routine use of advanced VTE therapies [64]. Therefore, the use of catheter-directed therapies during the current outbreak should be reserved for the most critical cases.

## 18. Additional Considerations

A lack of data makes it difficult to recommend transfusion thresholds in patients with COVID-19 that differ from those recommended for other critically ill patients. The prophylactic transfusion of platelets, use of fresh frozen plasma, fibrinogen, and prothrombin complex concentrate may be considered if invasive procedures are planned [18]. Lastly, patients requiring targeted temperature management often have prolongations of both PT and aPTT without evidence of bleeding diathesis [65]. Therefore, correction of coagulopathy in unselected patients without overt bleeding is not advisable.

## 19. Management of Bleeding That Occurs in COVID-19

Clinically overt bleeding is uncommon in patients with COVID-19. Bleeding in COVID-19-associated DIC requires support with blood products and should be managed as per local guidelines [66]. The guidelines for blood product transfusion are as follows: (a) maintain platelet count >50 × 10^9^/L in DIC patients with active bleeding or >20 × 10^9^/L in those with a high risk of bleeding or requiring invasive procedures, (b) fresh frozen plasma (15 to 25 mL/kg) in patients with active bleeding with either prolonged PT or aPTT ratios (>1.5 times normal) or decreased fibrinogen (<1.5 g/L), (c) fibrinogen concentrate, or cryoprecipitate in patients with persisting severe hypofibrinogenemia (<1.5 g/L), and (d) prothrombin complex concentrate if fresh frozen plasma transfusion is not possible. Tranexamic acid is not recommended for routine use in COVID-19-associated DIC.

## 20. Management of Patients with Thromboembolic Disease without COVID-19

The main management goals for patients with pre-existing or new onset thrombotic disease but without COVID-19 is to provide adequate antithrombotic protection, while minimizing physical contact between patients and healthcare workers. Outpatient management or early discharge for acute VTE is recommended whenever possible [57], and it is reasonable to plan early discharge after medication stabilization for low-risk ACS or PCI for high-risk ACS [67,68].

In general, pharmacotherapy in patients without COVID-19 but with thrombotic disease should be provided according to usual management plans. There is little evidence that antiplatelet agents or anticoagulants increase vulnerability to infection with COVID-19, or of developing severe COVID-19. Patients education on (a) self-monitoring of symptoms is important, and (b) visits to the emergency department for minor bleeding is discouraged.

Patients receiving vitamin K antagonist who need frequent INR checks face logistical challenges due to the lockdowns, with an increased risk of exposure to SARS-CoV-2 in public places. It is useful to consider alternatives such as extended INR testing intervals if previous INR values were stable [69]. Other alternatives include home-based INR checks (provided this is promptly enabled), drive-through INR testing, or switching to a DOAC or LMWHs when clinically appropriate [45].

Whenever switching of anticoagulant is planned, care should be taken to ensure the patients of affordability and accessibility of the agent. DOACs should be used with caution in the elderly (greater risk of bleeding, especially gastrointestinal bleeding) and those with acute kidney injury/renal impairment. Contraindications to treatment with DOACs include patients with mechanical heart valves, valvular atrial fibrillation (AF), antiphospholipid syndrome (APLS), concomitant use of drugs that inhibit cytochrome P450-family 3-subfamily A, and P-glycoprotein and patients who are pregnant or breastfeeding. Patient education on appropriate dietary habits while receiving vitamin K antagonists is also important. The use of LMWHs should be considered in cases where DOACs are not available or not approved by insurance providers.

## 21. International Travel and COVID-19

Multiple studies reported that environmental and physiological changes occur during routine commercial flights that could lead to mild hypoxia and gas expansion, and which can exacerbate chronic medical conditions or even induce acute in-flight medical events. Long-haul flights increase the risk of VET several fold. COVID-19 has at least two effects relevant to air travel. Unlike normal pneumonia, in which patients experience cough, chest discomfort and significant breathing difficulties, patients with COVID-19 pneumonia initially may not always experience such symptoms, causing a condition termed “silent” or “happy” hypoxia. This may be aggravated by the hypobaric cruising cabin altitude pressure. Silent hypoxia occurs because the virus only causes the air sacs to collapse to reduce oxygen levels without affecting the removal of carbon dioxide. It is important to detect hypoxia in these patients before they begin to experience dyspnea so that an early intervention can prevent the lungs from deteriorating further. Secondly, COVID-19 is associated with coagulopathy and endothelial damage resulting in VTE. As many economies return to normal, commercial aircrafts will resume operations whilst implementing preventative strategies. Pre- and on-board pulse oximetry screening can be used for early detection of silent hypoxia in unwell passengers boarding aircrafts. This is a simple procedure and can avoid on-flight emergencies. Identified risk factors may be accentuated by the procoagulant effects of undiagnosed COVID-19 that can increase the risk of VTE’s. The “new normal” pre- and post-travel consultation will need to take all these considerations into account. Travelers with known risk factors (e.g., obese, male, pregnant, smoking history, previous/family history of VTE) should consider appropriate thromboprophylaxis [70].

## 22. Public Health Considerations Related to Care for Thrombotic Disease

Governments have enacted mandatory home quarantine for all non-essential personnel in areas most affected by COVID-19. There are several issues to consider related to thrombotic disease at a community level:

1. Many patients become sedentary given the recommendations to remain at home, and are thus at increased risk for VTE [71,72,73]. This is particularly the case for the elderly and high-risk patients [74].
2. As daily routines continue to be disrupted, many will experience dietary changes (especially in daily intake of green vegetables, which are the major sources of vitamin K in Western diets) that can affect treatment with vitamin K antagonists. As the quarantine measures become even more restrictive, changes in physical activity, diet and vitamin K intake are likely to impact INR values further.
3. The COVID-19 pandemic has devastated the economy of many countries, with the United Nations estimating that COVID-19 could cost the world economy more than $1 trillion in 2020 [75,76]. This will negatively impact the ability of many patients to receive treatment for thrombotic diseases. Socioeconomic disadvantages are linked to higher rates of VTE and adverse outcomes [77,78].

## 23. Conclusions

More information and data are needed to better define thromboembolic disease due to de novo COVID-19 and differentiate it from pre-existing thrombotic disease to guide optimal management strategies. A large international registry is currently accruing data from COVID-19 patients with VTE [66,67] and another adjudicated prospective registry is incorporating COVID-19 outcomes with cardiovascular risks (CORONA-VTE registry; BWH Thrombosis Research Group). A multicenter, multinational ACS registry has also been initiated, in addition to the new American Heart Association registry for cardiovascular care and outcomes in these patients. Special attention should also be given to patients with pre-existing thromboembolic diseases and with limited access to care due constraints on access to the health care system. The guidance provided in this review for thrombotic disease and antithrombotic therapy during the COVID-19 pandemic (summarized in Table 2) should supplement rather than substitute for clinical decision making. Nuances in conversations between patients and practitioners should be considered when making appropriate patient-centered decisions. Thrombotic diseases may be existing factors or incident complications in patients with COVID-19. Mindful prescribing of preventive and therapeutic doses of antithrombotic agents will mitigate the potentially lethal thrombotic and hemorrhagic events in these high-risk patients. Collaboration between funding agencies, professional societies, patients, clinicians and investigators is needed to address current knowledge gaps on coagulopathies inpatients with COVID-19 patients. Lastly, as governments ease lockdowns, international travel will impact on how we manage and advise patients who remain at risk of, or who are recovering from, COVID-19.

## Figures and Tables

**Table 1:** Levels of D-Dimers in Patients with COVID-19.

Study and References	Levels in Non-Severe Patients (Confidence Interval	Levels in Severe Patients (Confidence Interval)	Significance Level (*p* Value)	Comments
Huang et al. (2020) [15]	0.5 mg/L	2.4 mg/L	*p* = 0.0042	ICU patients had significantly higher levels of D-dimer than non-ICU patients
Tang et al. (2020) [17]	0.61 (0.35−1.29)	2.12 (0.77−5.27)	*p* < 0.001	Overall mortality was 11.5%, the non-survivors revealed significantly higher D-dimer levels
Zhou et al. (2020) [25]	0.6 (0.3−1)	5.2 (1.5−21.1)	*p* < 0.0001	D-dimer levels > 1 μg/mL can help with early identification of patients with poor prognosis
Zhang et al. (2020) [27]	0.41 mg/L (0.15–0.69)	4.76 mg/L (2.99–11.9)	*p* < 0.001	D-dimer levels > 2.0 μg/mL on admission can predict in-hospital mortality in patients with COVID-19 and could be a therapeutic marker
Guan et al. (2020) [28]	43.2% with >0.5 mg/L	59.6% with >0.5 mg/L	N/A	D-dimer levels higher in those requiring ICU admission and invasive ventilation; statistical analysis not performed
Tu et al. (2020) [29]	Median 0.66 g/mL	Median 3.306 g/mL	*p* < 0.001	D-dimer levels were significantly higher in non-survivors

**Table 2:** Summary of management guidelines for thrombotic disease in patients with COVID-19.

*Status of Patient*	Management Recommendations
*Mild COVID-19*	Remain ambulatory at home Continue thromboprophylaxis for other indications at usual doses Switch to DOAC if available and affordable
*Moderate to severe COVID-19 without DIC*	D-dimer and platelet count checked every 2–3 days Prophylactic dose thromboprophylaxis if not strictly contraindicated Routine screening for VTE (despite elevated D-dimer levels (>1500 ng/mL) is not currently recommended.
*Moderate to severe COVID-19 with DIC*	Prophylactic dose thromboprophylaxis if not bleeding Patients already on chronic anticoagulation, dose is reduced after careful consideration of indication Patients on dual antiplatelet therapy should have dose adjustment based on serial platelet count
*Bleeding in COVID 19*	Uncommon but managed as per local guideline
*Thromboembolic disease without COVID-19*	Antithrombotic medication as per guideline. self-monitoring of symptoms is important visits to the emergency department for minor bleeding is discouraged Extended INR checks for patients receiving vit K antagonists LMWH or DOACs can be used if accessible and affordable

DOAC: Direct oral anticoagulants; DIC: Disseminated intravascular coagulation; VTE: Venous thromboembolism; INR: International normalized ratio; LMWH: Low-molecular weight heparin.

## Data Availability

Not applicable.
